# Medial Unicompartmental Knee Arthroplasty After Previous Patellectomy: A Case-Based Review and Technical Considerations

**DOI:** 10.7759/cureus.117852

**Published:** 2026-10-13

**Authors:** Alexandre Castro, Paula Sousa, Henrique Sousa, Ricardo Santos Pereira, Miguel Quesado

**Affiliations:** 1 Orthopedics and Traumatology, Unidade Local de Saúde Entre o Douro e Vouga, Santa Maria da Feira, PRT; 2 Orthopedics and Traumatology, Centro Hospitalar Vila Nova de Gaia/Espinho, Vila Nova de Gaia, PRT

**Keywords:** extensor mechanism, knee osteoarthritis, patellectomy, surgical technique, unicompartmental knee arthroplasty

## Abstract

The patella plays an essential role in optimizing the biomechanics of the knee extensor mechanism. Patellectomy disrupts force transmission within the extensor apparatus, leading to diminished extension strength, altered joint stability, and a higher risk of developing tibiofemoral osteoarthritis. Surgical treatment of degenerative changes in patellectomized knees remains challenging, and evidence regarding the use of unicompartmental knee arthroplasty (UKA) in these patients is limited.

We present the case of a 66-year-old man who developed persistent anteromedial knee pain and functional impairment more than two decades after total patellectomy. Imaging studies demonstrated isolated osteoarthritis of the medial compartment. The patient underwent medial mobile-bearing UKA using the standard anteromedial surgical approach. Postoperative management included early mobilization and gradual progression to full weight-bearing. At the 36-month follow-up, the patient reported no pain and achieved a knee range of motion of 0-140°, with quadriceps strength comparable to that of the contralateral side. Radiographs demonstrated appropriate implant positioning without evidence of loosening, misalignment, or progression of degenerative changes in the remaining compartments. This case suggests that patellectomy may not be a contraindication to UKA in carefully selected patients with isolated medial disease and intact ligamentous stability and that UKA may provide durable mid-term outcomes.

## Introduction

The patella contributes significantly to knee biomechanics. By increasing the lever arm of the quadriceps muscle, it enhances the mechanical efficiency of the extensor mechanism. Patellectomy results in substantial biomechanical alterations, including a reduction in the quadriceps moment arm of approximately 13-30% and a decline in extensor mechanism efficiency of up to 40%. Consequently, patients may experience reduced extension strength, extension lag, and an increased risk of anteroposterior instability [1,2].

Although total patellectomy is currently considered a salvage procedure and is performed infrequently, individuals who have undergone the surgery in the past may later develop symptomatic knee osteoarthritis requiring surgical treatment. Patellectomy modifies the force distribution across the knee joint, increases contact stresses in the tibiofemoral articulation, and alters normal kinematics. These changes may contribute to mechanical overload and accelerated wear and potentially influence outcomes following total knee arthroplasty (TKA) [3,4].

In recent years, the utilization of unicompartmental knee arthroplasty (UKA) has increased, partly because of broader indications and improved implant designs [5]. Nevertheless, published data on UKA in patients with a prior patellectomy remain extremely limited and consist primarily of isolated case reports [6-8].

To our knowledge, reports of medial mobile-bearing UKA in patellectomized patients with mid-term follow-up remain scarce.

The purpose of this report was to describe the management of medial compartment osteoarthritis in a patellectomized patient treated with UKA. The surgical technique, clinical outcomes, and possible biomechanical advantages of this approach are discussed.

The patient was informed that data concerning the case would be submitted for publication and provided consent.

## Case presentation

Patient selection

Appropriate patient selection is essential to achieve optimal outcomes when considering UKA in patients with a prior patellectomy. Indications for this procedure include the presence of isolated medial compartment osteoarthritis, with preservation of the lateral compartment, as well as intact anterior and posterior cruciate ligaments. A correctable varus deformity and the absence of significant ligamentous instability are also fundamental requirements. In this context, UKA may offer biomechanical advantages by preserving native ligamentous structures and maintaining more physiological joint kinematics.

Conversely, contraindications include multicompartmental osteoarthritis, ligament insufficiency, fixed deformities, and significant anteroposterior instability. Patients with inflammatory arthropathies or advanced degenerative changes involving multiple compartments are not suitable candidates for this technique, as these conditions may compromise implant survival and functional outcomes.

A thorough preoperative evaluation is critical to ensure an appropriate indication. A detailed medical history should include prior surgical procedures, particularly the indication and timing of patellectomy, as well as the progression of symptoms and functional limitations. Clinical examination must assess limb alignment, range of motion, and, most importantly, ligamentous stability. Special attention should be given to detecting anteroposterior instability, which may be more subtle in patellectomized patients due to altered biomechanics of the extensor mechanism.

Imaging studies play a central role in confirming the indication for UKA. Standard radiographs, including weight-bearing anteroposterior, lateral, and long-leg alignment views, are essential to evaluate joint space narrowing, deformity, and overall limb alignment. Magnetic resonance imaging may be useful to further assess cartilage status, confirm preservation of the lateral compartment, and evaluate the integrity of the cruciate and collateral ligaments. This comprehensive assessment allows accurate identification of suitable candidates and helps minimize the risk of inappropriate indications.

Clinical presentation

A 66-year-old man presented to our hospital with persistent anteromedial left knee pain and progressive deterioration of walking ability.

He had a history of total patellectomy performed more than 20 years earlier following a patellar fracture. His medical history included high blood pressure and dyslipidemia.

Physical examination revealed varus alignment of both knees, diffuse tenderness along the medial joint line, absence of joint effusion, and no signs of instability. Valgus and varus stress tests in full extension and at 30° of flexion demonstrated stability. There was no posterior sag. The Lachman and anterior drawer tests were also negative. The extensor mechanism was intact, and quadriceps muscle strength was graded as 5/5 on the Medical Research Council scale [9]. Knee range of motion was limited to 0-120°.

Plain radiographs, including anteroposterior, lateral, Merchant, and full-length weight-bearing views, revealed an absent patella, medial compartment narrowing with a mild, correctable varus deformity of approximately 4°, and medial joint line osteophytes (Figures 1, 2). 

**Figure 1:**
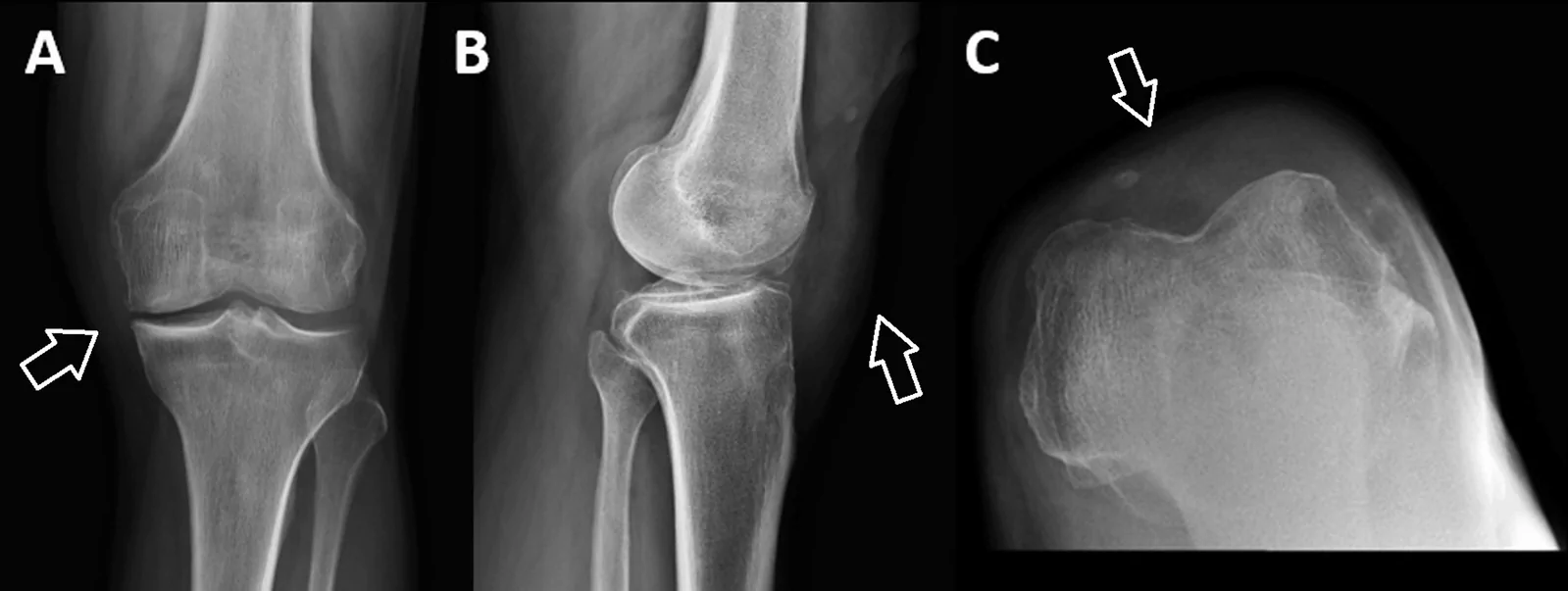
Weight-bearing anteroposterior (A), lateral (B), and Merchant (patellofemoral) (C) radiographic views of the left knee demonstrating absence of the patella, medial compartment joint space narrowing, and the presence of joint-line osteophytes.

**Figure 2:**
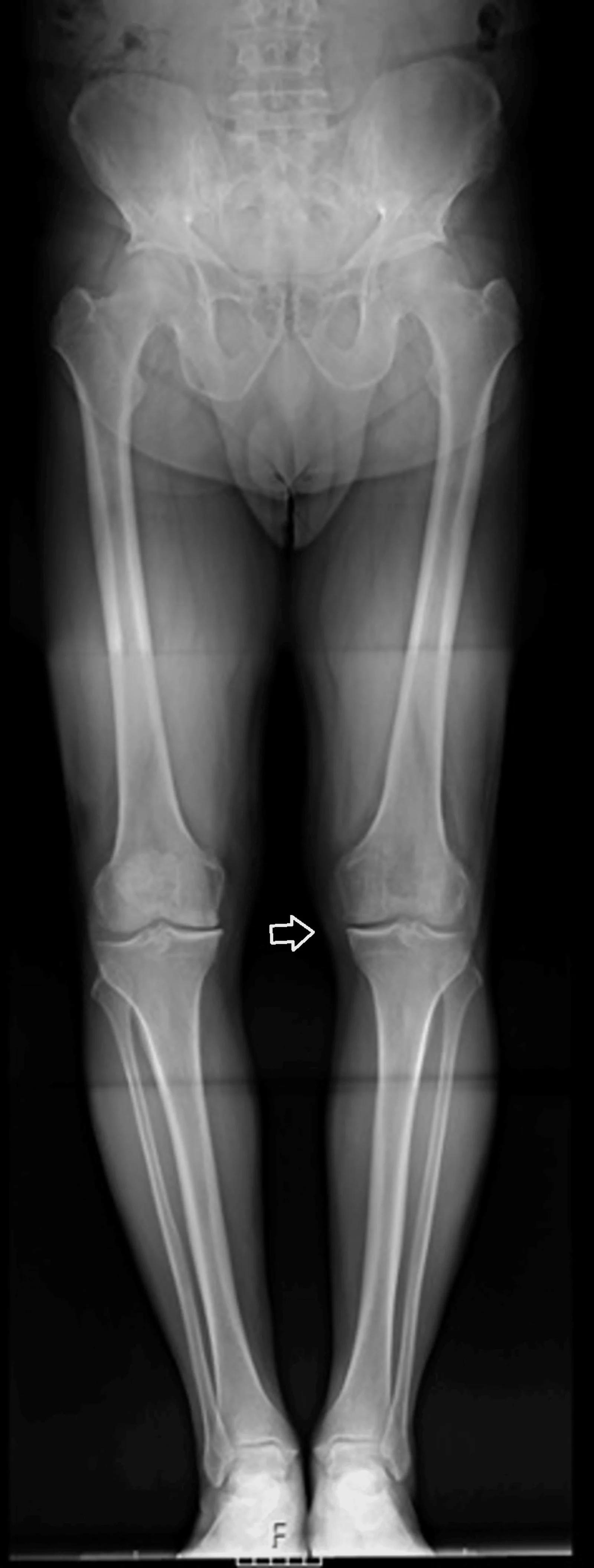
Weight-bearing long-leg alignment radiographs demonstrating varus malalignment of the mechanical axis measuring 4°.

Magnetic resonance imaging (Figure 3) confirmed advanced osteoarthritis of the medial knee compartment, characterized by osteophyte formation, diffuse grade IV cartilage loss, and a complex medial meniscus tear. The lateral compartment was preserved. The cruciate and collateral ligaments were intact and showed normal morphology. The quadriceps and patellar tendons were continuous, with mild hypointensity on T1-weighted images, consistent with the sequelae of prior surgical intervention. 

**Figure 3:**
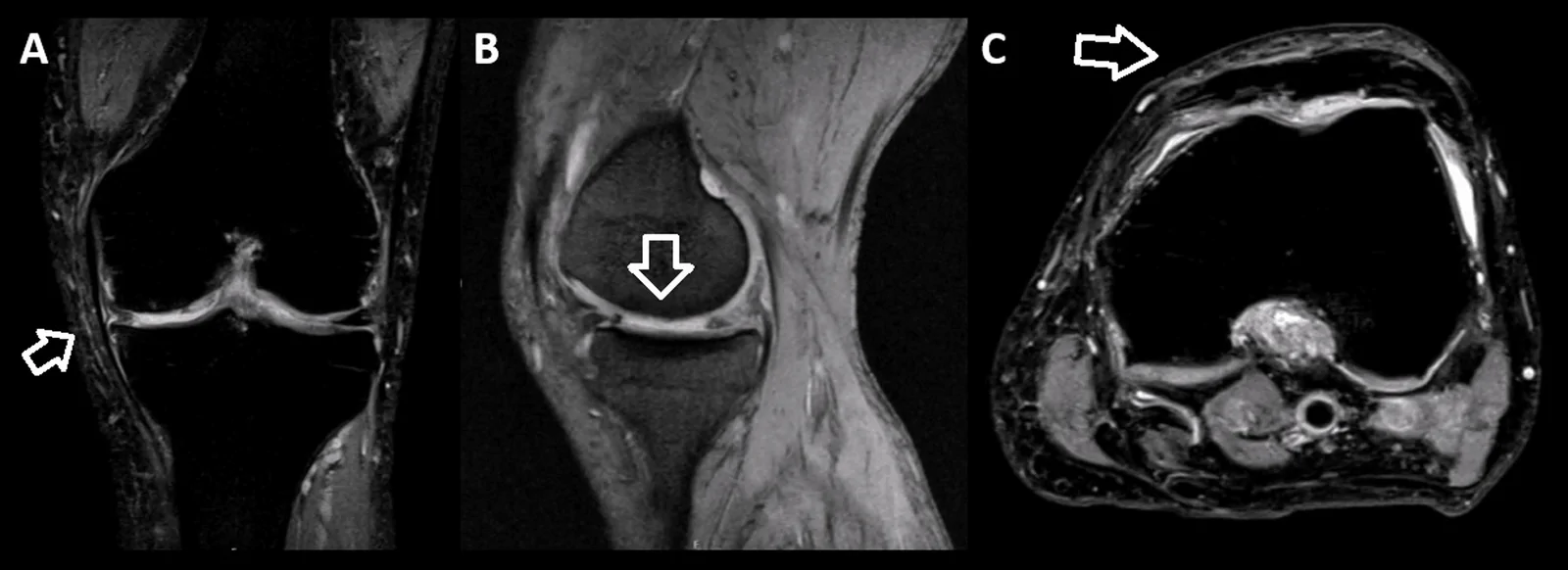
Magnetic resonance imaging in coronal (A), sagittal (B), and axial (C) views confirming advanced osteoarthritis of the medial knee compartment, characterized by osteophyte formation, diffuse grade IV cartilage loss, and a complex medial meniscus tear. The lateral compartment was preserved. The cruciate ligaments, as well as the medial and lateral collateral ligaments, were intact and demonstrated normal morphology. The quadriceps and patellar tendons were continuous, with mild hypointensity on T1-weighted images, consistent with sequelae of prior surgical intervention.

Preoperative functional assessment showed a Knee Society Score [10] of 55/100, indicating poor function with significant symptoms consistent with moderate to severe functional limitation. The patient underwent mobile-bearing UKA (Oxford Partial Knee, Zimmer Biomet).

Surgical technique

The surgical technique is described below, and the key technical pearls and pitfalls are summarized in Table 1.

**Table 1:** Key technical points (pearls and pitfalls) ACL: anterior cruciate ligament; PCL: posterior cruciate ligament; AP: anteroposterior.

Pearls
Strict patient selection is essential
Preservation of ligament integrity, ACL and PCL, is critical
Avoid overcorrection of varus deformity
Assess AP stability dynamically with the trial bearing throughout the full range of motion.
Pitfalls
Undiagnosed ligament insufficiency
Overcorrection leading to lateral compartment overload
Inadequate assessment of compartment involvement

Positioning

A thigh tourniquet was applied. The patient was positioned with the hip flexed approximately 30°. The leg was supported at the thigh, allowing the limb to hang freely and enabling a full knee range of motion.

Approach

A standard anteromedial approach was used. Careful soft tissue handling was essential due to previous surgical alterations.

Intraoperative Assessment

Isolated medial compartment disease was confirmed. The integrity of the cruciate and collateral ligaments and the condition of the lateral compartment and trochlear cartilage were assessed. Particular attention was paid to anteroposterior tibiofemoral stability, as patellectomy may reduce the dynamic stabilizing effect of the extensor mechanism. Any evidence of significant instability should prompt reconsideration of the indication for UKA.

Bone Preparation

Tibial and femoral cuts were performed according to the standard UKA technique (Oxford Partial Knee, Zimmer Biomet). Alignment was planned to avoid overcorrection of the preexisting deformity. Restoration of the patient's constitutional alignment was preferred over neutral mechanical alignment to avoid excessive correction that could overload the lateral compartment and compromise ligament balance.

Implant Positioning

A mobile-bearing prosthesis (Oxford Partial Knee, Zimmer Biomet) was implanted. Proper sizing and positioning were critical to ensure stability and avoid bearing dislocation. A mobile-bearing design was preferred to preserve native knee kinematics while maintaining low implant constraint, provided that adequate ligament balance was achieved.

Stability Assessment

Intraoperative testing was performed to confirm balanced flexion and extension gaps. Gap balancing was carefully assessed to avoid excessive laxity, which may increase the risk of bearing instability. Dynamic assessment with the trial bearing during repeated flexion-extension cycles was performed to confirm stable bearing tracking and exclude excessive laxity before final implantation.

Postoperative Management

Early mobilization was initiated on the first postoperative day, followed by progressive weight bearing as tolerated and structured rehabilitation focusing on quadriceps strengthening and gait restoration. The patient was discharged on postoperative day one (Figure 4).

**Figure 4:**
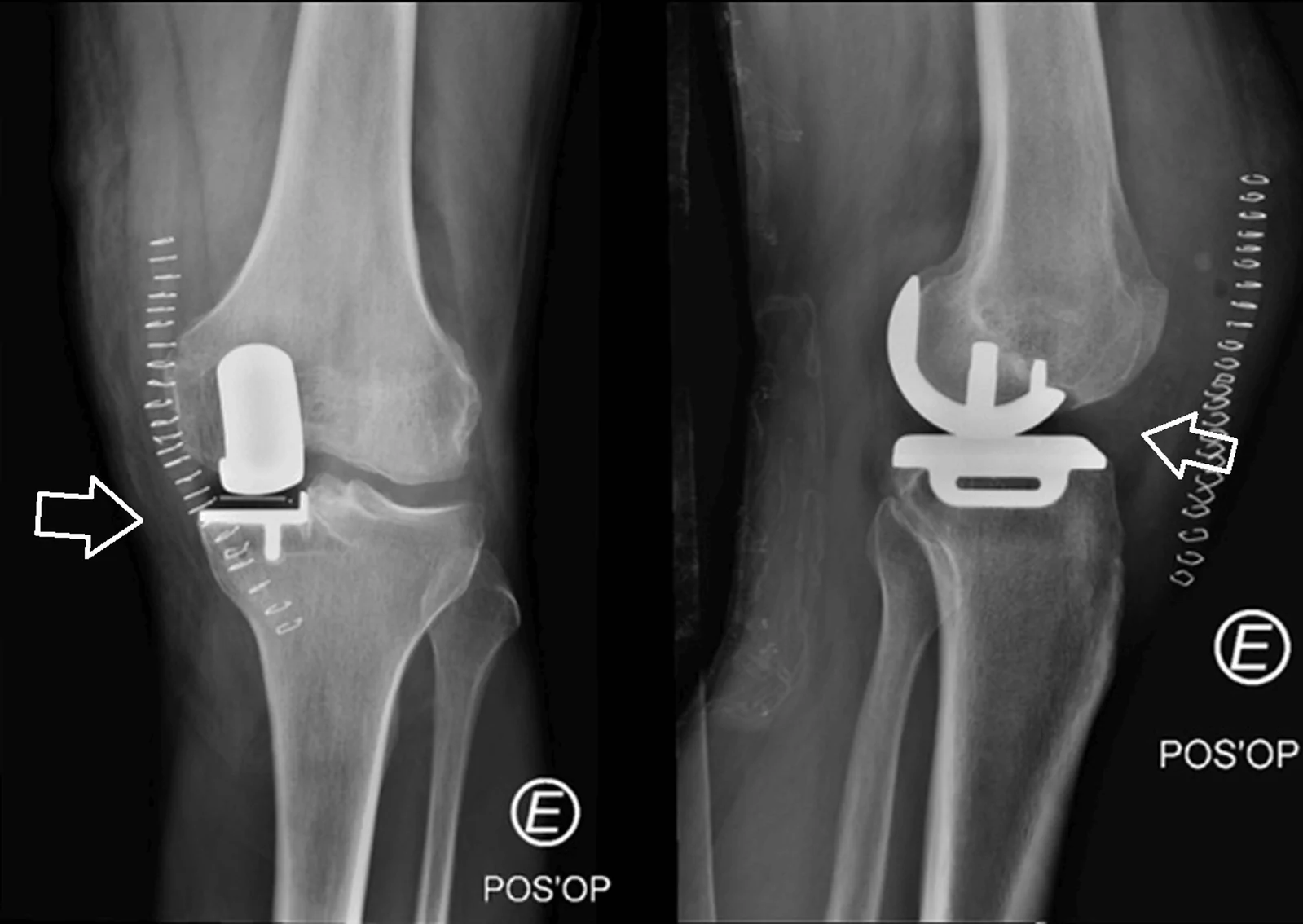
Postoperative anteroposterior and lateral radiographs of the left knee demonstrating unicompartmental medial compartment replacement.

At the one-month follow-up, the patient tolerated full weight bearing without complications, and the rehabilitation program was continued with an emphasis on muscle strengthening and restoration of full load-bearing capacity. Two months postoperatively, the active and passive knee range of motion was 0-140°, with mild residual pain associated with a slight reduction in flexion and extension strength.

At the 36-month postoperative follow-up, the knee range of motion remained 0-140°, with flexion and extension strength comparable to that of the contralateral limb. Anteroposterior and lateral radiographs of the left knee after medial UKA showed that the implant remained well aligned, with no subsidence, malposition, or loosening (Figure 5). 

**Figure 5:**
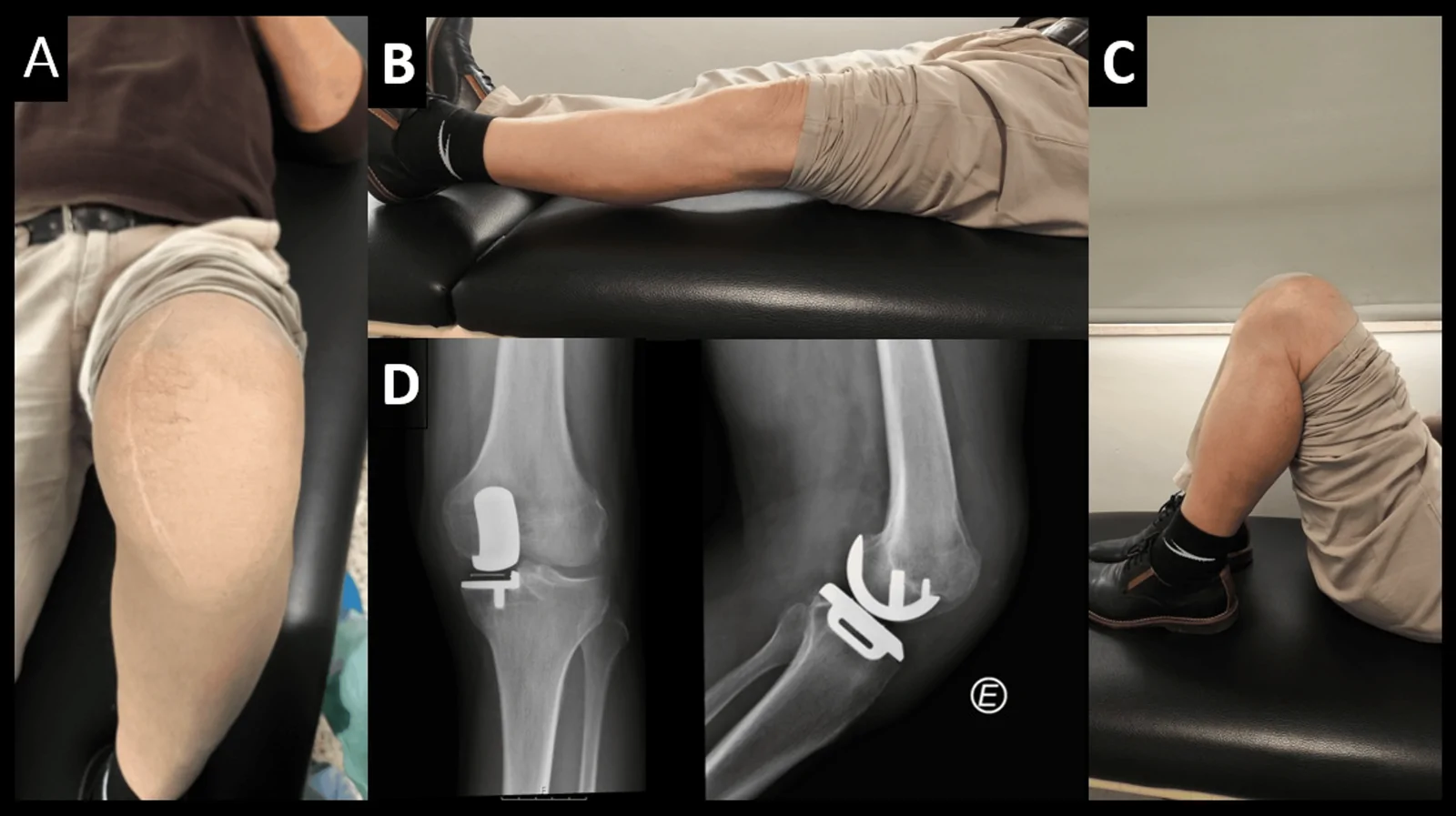
Clinical photographs of the left knee three years after medial unicompartmental arthroplasty (A-C), showing the surgical incision and active range of motion in full extension and flexion. Three-year postoperative anteroposterior and lateral radiographs of the left knee after medial unicompartmental replacement (D), showing a well-aligned implant with no subsidence, malposition, or loosening.

## Discussion

Patellectomy is rarely performed in modern orthopedic practice. Consequently, there is limited guidance regarding optimal treatment strategies for patients who later develop degenerative knee disease [1,11].

Patellectomy leads to multiple biomechanical alterations of the knee joint. These include reduced efficiency of the quadriceps muscle, increased contact pressure within the tibiofemoral joint, and compromised anteroposterior stability. Because of these changes, concerns have been raised about the reliability of arthroplasty procedures in patellectomized knees, and the reported outcomes following TKA have been inconsistent [4,11].

Previous studies evaluating TKA in patients without a patella reported variable functional results, with postoperative instability representing one of the most frequently described complications [3,4,11]. In contrast, UKA preserves the cruciate ligaments, particularly the anterior cruciate ligament, which plays a key role in maintaining sagittal plane stability. Retention of these stabilizing structures may offer biomechanical advantages for appropriately selected patellectomized patients [6-8].

Although evidence remains limited, the few reports describing UKA in patellectomized patients have demonstrated encouraging results in individuals with isolated medial compartment degeneration and intact ligamentous structures [6-8].

The present case provides additional support that a history of patellectomy does not necessarily preclude the use of UKA. UKA may provide favorable functional outcomes while maintaining physiological joint mechanics in carefully selected patients with preserved ligamentous stability and localized compartment disease. Compared with TKA, its potential advantages include reduced surgical invasiveness, shorter hospitalization, and faster recovery [12].

Nevertheless, the findings of this report should be interpreted cautiously given the single-patient design and relatively limited follow-up duration. Further studies are necessary to determine the role of UKA in this clinical context.

## Conclusions

A review of the available literature suggests that successful outcomes of UKA after patellectomy are consistently associated with strict patient selection criteria, including isolated unicompartmental disease, preserved cruciate ligament integrity, and the absence of significant instability. In contrast, studies evaluating TKA in patellectomized patients report less predictable outcomes, particularly related to anteroposterior instability and extensor mechanism inefficiency. These findings support the hypothesis that preservation of native ligamentous structures in UKA may be particularly advantageous in this population. Larger studies with longer follow-up periods are required to confirm these results.
